# Acoustic Cardiography (ACG) for Left Ventricular Ejection Time (LVET) Monitoring in Preeclampsia Risk Prediction

**DOI:** 10.1002/clc.70210

**Published:** 2025-09-14

**Authors:** Chunping Tang, Xinxin Zhang, Miao Wang, Yiyuan Xiong, Yingxia Zhu, Qiong Huang, Ningtian Zhou

**Affiliations:** ^1^ Department of Cardiology The First Affiliated Hospital with Nanjing Medical University Nanjing China; ^2^ Department of Obstetrics The Affiliated Kezhou People's Hospital with Nanjing Medical University Kezhou China; ^3^ Department of General Practice The First Affiliated Hospital with Nanjing Medical University Nanjing China

**Keywords:** acoustic cardiography, left ventricular ejection time, preeclampsia, risk prediction

## Abstract

**Background:**

Preeclampsia (PE), a leading cause of maternal morbidity, lacks reliable early biomarkers. This study evaluates acoustic cardiography (ACG) for noninvasive left ventricular ejection time (LVET) monitoring and its predictive value in PE.

**Methods:**

In an observational case‐control study, 59 pregnant women (28 controls, 31 PE cases) underwent synchronized ECG‐phonocardiogram (PCG) monitoring using AI‐driven devices. LVET, Q2S2Max, and hemodynamic parameters were analyzed.

**Hypothesis:**

ACG predict PE risk via LVET monitoring.

**Results:**

Significantly prolonged LVET in the PE group (320.28 ± 26.79 ms vs. 301.32 ± 35.42 ms, *p* = 0.026), correlating with increased cardiac afterload. ROC analysis revealed moderate diagnostic efficacy for LVET alone (AUC = 0.658, sensitivity 72.4%, specificity 57.1%). Combining LVET with hypertension history enhanced performance (AUC = 0.776, specificity 77.8%), reducing false positives. Elevated Q2S2Max in PE (426.10 ± 29.46 vs. 403.96 ± 33.28, *p* = 0.010) indicated vascular stiffness, suggesting early vascular‐cardiac coupling dysfunction.

**Conclusions:**

ACG‐derived parameters, integrated with clinical risk factors, demonstrated cost‐effective, dynamic monitoring potential for early PE detection, particularly in resource‐limited settings. While limited by sample size and single‐center design, this study highlights ACG as a promising tool for cardiovascular risk stratification in pregnancy, warranting further validation in larger cohorts.

## Graphical abstract

ACG‐derived parameters (LVET), integrated with clinical risk factors, demonstrated dynamic monitoring potential for early Preeclampsia detection, particularly in resource‐limited settings.

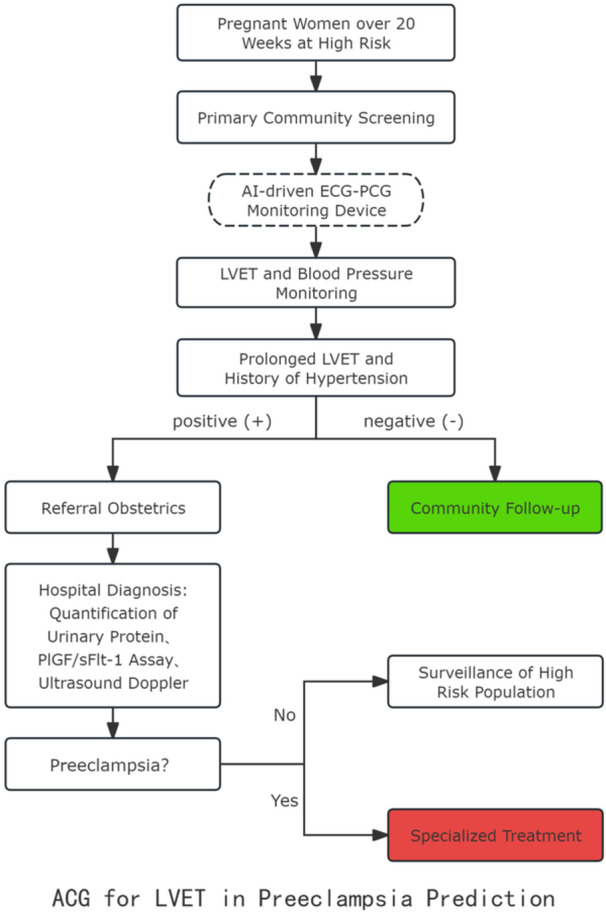

## 1 Introduction

Preeclampsia (PE), a pregnancy‐specific multisystem disorder, is one of the leading causes of maternal and perinatal mortalities worldwide. Globally, the incidence ranges from 5% to 10% [1]. However, in Xinjiang, China, owing to vast geographical areas, uneven distribution of medical resources, and ethnic diversity, the incidence of PE is significantly higher than the national average (reaching 10.2%), with severe cases accounting for 35% of all cases [2]. This increases the risk of preterm birth, placental abruption, fetal growth restriction, and other serious complications. Current clinical diagnosis primarily relies on elevated blood pressure ( ≥ 140/90 mmHg) and proteinuria ( ≥ 300 mg/24 h), symptoms often manifest in the middle‐to‐late stages of the disease, missing the optimal intervention window [3]. Although laboratory biomarkers such as the soluble fms‐like tyrosine kinase‐1 (sFlt‐1) to placental growth factor (PlGF) ratio show high predictive value [4, 5], their high cost and technical complexity limit their adoption in primary healthcare settings. Thus, exploring noninvasive, real‐time, and dynamic early warning indicators is of significant clinical importance.

The core pathological mechanism of PE involves systemic vascular endothelial dysfunction and placental ischemia, triggering systemic inflammation and oxidative stress, ultimately leading to hypertension and multiorgan damage [6, 7]. Recent studies have highlighted the critical role of the cardiovascular system in PE: pregnancy increases blood volume by 50%–60% and cardiac output by 30%–50%, but PE patients experience abnormally elevated peripheral vascular resistance and myocardial remodeling, significantly increasing cardiac afterload [8]. Research indicates that women with early onset PE (before 34 weeks) often exhibit mild‐to‐moderate diastolic dysfunction, while the left ventricular ejection fraction (LVEF) remains normal or mildly elevated in early stage PE, suggesting that traditional cardiac metrics (e.g., LVEF) lack sensitivity to early pathological changes [9]. In contrast, left ventricular ejection time (LVET), a sensitive parameter reflecting cardiac afterload, can detect hemodynamic abnormalities earlier using systolic time interval (STI) analysis [10]. Traditionally, LVET is measured via echocardiography or cardiac catheterization, but the former requires specialized operators and the latter is invasive, both impractical for dynamic monitoring.

Acoustic cardiography (ACG), synchronous electrocardiography (ECG), and heart sound monitoring technology offer an innovative solution for cardiac monitoring in pregnancy. By analyzing the temporal relationship between ECG signals (QRS complex) and heart sound vibrations (S1/S2), ACG enables noninvasive, real‐time LVET calculation, the time interval from the opening to the closing of the aortic valve during systole (measured in milliseconds). Compared to traditional echocardiography, ACG combines portability (device weight < 13 g) and dynamic monitoring capabilities (continuous recording ≥ 24 h), demonstrating superiority in managing cardiovascular diseases, such as heart failure and valvulopathy. Studies confirm that AI models based on ECG–heart sound multimodal data achieve an AUC of 0.92 for detecting left ventricular systolic dysfunction (LVSD), validating its clinical reliability [11]. Nevertheless, its application under pregnancy‐specific conditions, such as PE, remains unexplored.

In this study, we proved that ACG technology is reliable for LVET monitoring and may serve as an independent predictive biomarker for PE, and its integration with traditional risk factors (e.g., history of hypertension) could significantly improve early diagnostic efficacy.

## 2 Materials and Methods

### 2.1 Study Design and Ethical Compliance

This observational case‐control study was conducted in the obstetrics outpatient and inpatient departments of Xinjiang Kezhou People's Hospital between January 2022 and June 2023. The protocol was approved by the hospital's Ethics Committee (Approval No.: 2023‐02‐03) and written informed consent was obtained from all participants. The study strictly adhered to the principles of the Declaration of Helsinki and participant privacy was protected through data anonymization.

### 2.2 Study Population

#### 2.2.1 Inclusion Criteria

Singleton pregnancy, gestational age ≥ 20 weeks, age 18–45 years, voluntary participation, and completion of all required examinations.

#### 2.2.2 Exclusion Criteria

Congenital heart disease, thyroid dysfunction, chronic kidney disease, autoimmune disorders, pre‐existing hypertension or diabetes, or use of cardiac‐affecting medications (e.g., beta‐blockers).

Participants were classified according to the 2020 Guidelines for Diagnosis and Management of Hypertensive Disorders in Pregnancy [12]. A total of 59 pregnant women were enrolled and divided into two groups: non‐PE (*n* = 28) and PE (*n* = 31). The PE group was further stratified into mild (blood pressure ≥ 140/90 mmHg with proteinuria) and severe (blood pressure ≥ 160/110 mmHg or end‐organ damage).

#### 2.2.3 Baseline Data Collection

Data on age, pre‐pregnancy weight, prenatal weight, obstetric history, and family history of hypertension were collected via electronic medical records and questionnaires. Height and blood pressure (averaged over three measurements at 5‐min intervals) were recorded at enrollment, and the body mass index (BMI) was calculated.

### 2.3 Multi‐Modal ECG–PCG Data Acquisition and Intelligent Analysis System

The AI‐driven ECG–PCG synchronous monitoring device (WENXIN device, Wenxin Tech, Fuzhou, China) employed in this study integrates electromechanical coupling principles to collect ECG and phonocardiogram (PCG) signals via high‐precision sensors, with multimodal feature fusion and pathological classification achieved using a deep residual network (ResNet). The WENXIN device (Wenxin Tech, Fuzhou, China) was used for acoustic cardiac monitoring.

#### 2.3.1 Signal Synchronization and Preprocessing

Disposable patch electrodes were placed in the left fourth intercostal space (aortic valve auscultation area) to synchronously record the ECG (sampling rate ≥ 500 Hz) and PCG (frequency range 20–1000 Hz). ECG signals were filtered with a 0.05–150 Hz bandpass to remove baseline drift, while PCG signals underwent wavelet packet decomposition (Daubechies 6 basis function) for noise suppression and enhanced the time‐frequency resolution of the S1/S2 components.

#### 2.3.2 Intelligent Signal Fusion and Key Parameter Extraction

**FIGURE 1:**
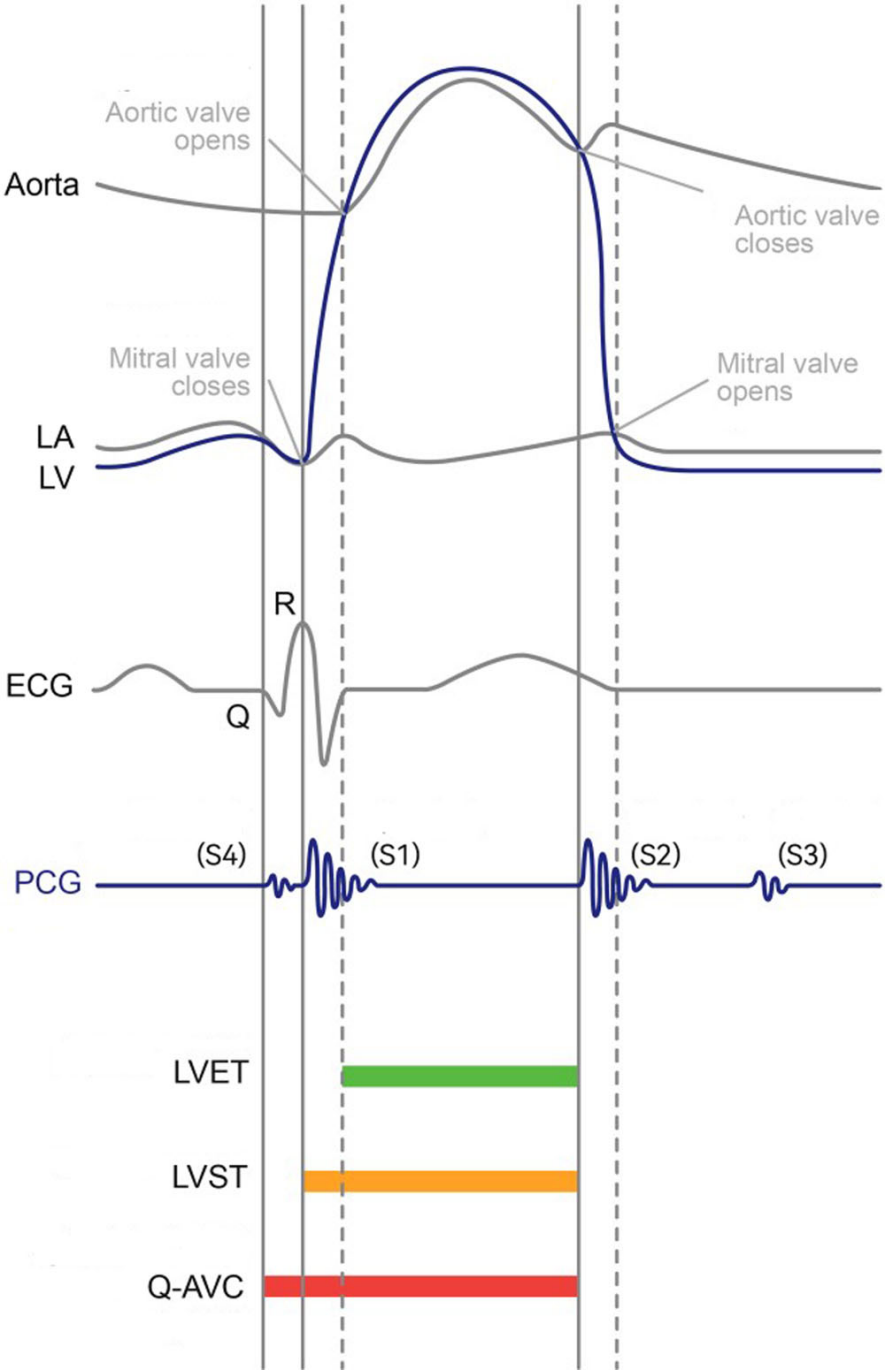
Cardiac sounds and electrocardiogram illustration. LA: Left Atrium, LV: Left Ventricular, LVET: Left Ventricular Ejection Time, LVST: Left Ventricular Systolic Time, Q‐AVC: Time from point Q to Aortic Valve Closure.

Using the QRS complex onset as a temporal reference, mel‐frequency cepstral coefficients (MFCCs) were extracted from the PCG signals. The dynamic time warping (DTW) algorithm identified mitral valve closure (S1) and aortic valve opening (S2) events. Bluetooth 5.0 enabled millisecond‐level synchronization (error < 1 ms) between ECG and PCG signals, dynamically calibrating mechanical event timing within cardiac cycles (Figure 1). The multi‐modal features include the following [13]:

- **LVET**: Measured from the end of S1 to the peak of the second heart sound(S2), representing the time period from aortic valve opening to aortic valve closure
- **Q2S2Max**: Ratio of S2 peak amplitude to QRS amplitude (dimensionless, indicating aortic valve closure strength).
- **EMAT**: Electromechanical activation time, The interval in ms from the onset of the QRS to the point of peak intensity of S1.

Building on prior technological advancements, this study establishes a hemodynamic evaluation system that integrates multimodal fusion. By resolving the S1–S2 intervals at a resolution of ± 1 ms and employing a Part II‐optimized QRS detection algorithm. The model utilized a ResNet architecture with cross‐layer feature reuse (eight residual blocks, 3 × 3 kernels). Input layers received ECG spectrograms (STFT‐generated, 640×480 pixels) and PCG MFCCs (40‐dimensional vectors), with dual‐channel attention mechanisms dynamically weighting electro‐mechanical signal contributions (weight ratio 1.23:1). Validated on the EPHNOGRAM database, the model achieved a mean absolute error (MAE) of 8.2 ms for LVET prediction, reducing discrepancies by 37% compared to traditional ultrasound.

Part I's optimized signal quality (SQI = 0.89) ensured stable STFT spectrograms (0.5 Hz frequency resolution), while Part II's time‐frequency fusion framework supported MFCC extraction (wavelet scale 128 × 512 translation).

#### 2.3.3 Standardized Protocol and Quality Control

- **Positioning**: The participants rested in the left lateral decubitus position for 5 min before sensor placement.
- **Real‐time QC**: AI algorithms monitored signal quality (SQI > 0.8 for validity), rejected motion artifacts (accelerometer threshold > 0.5 g), and baseline drift (amplitude > 1 mV).
- **Dynamic Calibration**: Automatic gain adjustment ( ± 30%) compensated for gestational chest wall thickening (3–5 mm increase in late pregnancy), maintaining S1/S2 amplitude stability (CV < 8%).

#### 2.3.4 Echocardiographic Validation

Two blinded operators used a GE Vivid E95 ultrasound system (M5S probe, 1.5–4.6 MHz) to measure the left ventricular ejection fraction (LVEF) using the Teichholz method. Triplicate measurements yielded an intraclass correlation coefficient (ICC) of 0.91.

#### 2.3.5 Supplementary Metrics

- **Ambulatory Blood Pressure Monitoring (ABPM)**: Omron HEM‐7136 recorded blood pressure every 30 min.
- **Laboratory Tests**: 24‐h urinary protein, serum creatinine, uric acid, and liver enzyme levels

### 2.4 Statistical Analysis

Data were analyzed using SPSS 25.0 and MedCalc 20.0. Normally distributed continuous variables are expressed as mean ± SD (independent samples *t*‐test), while non‐normal data are expressed as median (IQR) (Mann‐Whitney U test). Categorical variables are described as frequency (%) (chi‐square or Fisher's exact test). The sample size was determined using effect size calculations for the independent t‐tests.

#### 2.4.1 Multivariate Analysis

Variables with *p* < 0.1 in univariate analysis (LVET, hypertension history, heart rate) were included in a logistic regression model (forward stepwise selection) to identify independent predictors, with odds ratios (ORs) and 95% confidence intervals (CIs) calculated.

#### 2.4.2 Diagnostic Performance

Receiver operating characteristic (ROC) curves evaluated AUC, sensitivity, specificity, and optimal cutoff values. Combined diagnostic models generated prediction probabilities via logistic regression, with DeLong's test comparing the AUC differences. Statistical significance was set at α = 0.05 (two‐tailed).

## 3 Result

### 3.1 Clinical Characteristics and Group Comparison

A total of 59 pregnant women were included in this study. Among them, 28 cases (47.5%) belonged to the control group (normal blood pressure and no complications) and 31 cases (52.5%) belonged to the PE group. There was no significant difference in baseline demographic characteristics between the two groups: age (28.6 ± 4.2 years vs. 29.1 ± 5.0 years, *p* = 0.674), height (163.5 ± 4.5 cm vs. 160.0 ± 3.7 cm, *p* = 0.335), gestational age (32.4 ± 3.1 weeks vs. 31.8 ± 3.5 weeks, *p* = 0.482), and pre‐pregnancy BMI (30.1 ± 3.0 kg/m² vs. 31.6 ± 3.4 kg/m², *p* = 0.418).

However, the PE group showed characteristic cardiovascular functional changes: LVET was significantly prolonged (320.28 ± 26.79 ms vs. 301.32 ± 35.42 ms, *p* = 0.026), suggesting increased left ventricular ejection resistance; Q2S2Max amplitude increased (426.10 ± 29.46 vs. 403.96 ± 33.28, *p* = 0.010), reflecting enhanced aortic valve closure impulse; AI LVST was prolonged (342.00 vs. 320.50 ms, *p* = 0.045), consistent with the prolongation of systolic time caused by increased afterload. Moreover, the proportion of history of hypertension was higher in the PE group (3.57% vs. 29.03%, *p* = 0.013). The clinical characteristics of the two groups are shown in Table 1.

**TABLE 1:** Clinical characteristics.

Parameters	Negative control (*n* = 28)	Preeclampsia (*n* = 31)	P value
Age (years)	28.6 ± 4.2	29.1 ± 5.0	0.674
Height (cm)	163.5 ± 4.5	160.0 ± 3.7	0.335
Gestational weeks	32.4 ± 3.1	31.8 ± 3.5	0.482
Pre‐pregnancy BMI (kg/m²)	30.1 ± 3.0	31.6 ± 3.4	0.418
LVEF (%)	62.8 ± 2.6	64.8 ± 3.5	0.021*
LVET (ms)	301 ± 35.42	320 ± 26.79	0.010*
LVST(ms)	320.5 ± 20.5	342.0 ± 16.5	0.045*
Q2S2Max (mV)	403.96 ± 33.28	426.1 ± 29.46	0.010*
Hypertension (%)	3.57	29.03	0.013*

Abbreviations: LVEF, left ventricular ejection fraction; LVET, Left Ventricular Ejection Time; Q2S2Max, Maximum amplitude of second heart sound.

### 3.2 Diagnostic Efficacy of LVET as a Single Factor

**FIGURE 2:**
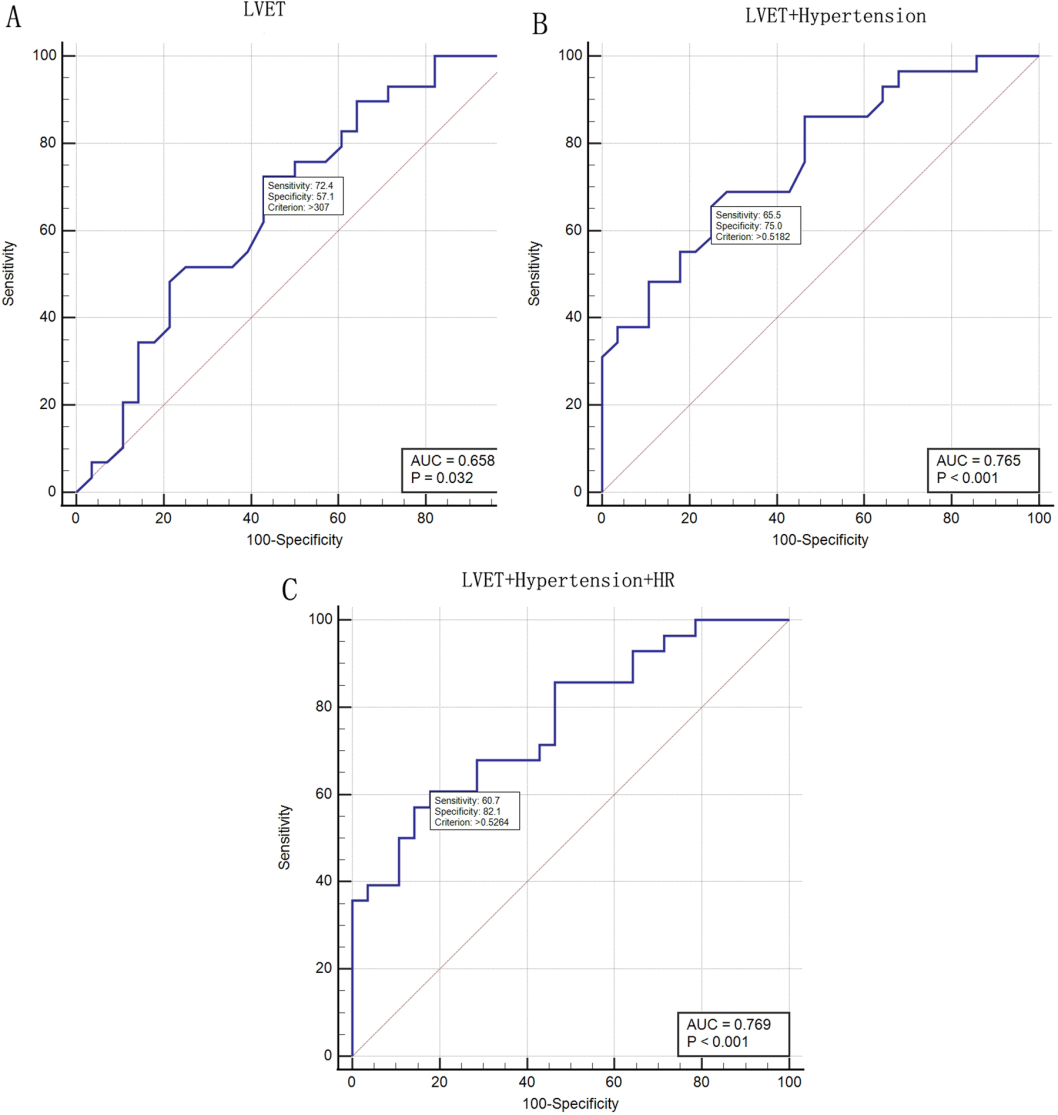
ROC of three diagnostic models. (A) LVET, (B) LVET+ Hypertension, (C) LVET+ Hypertension+ HR.

ROC curve analysis showed that the single‐factor diagnostic efficacy of LVET for PE was moderate (AUC = 0.658, 95% CI: 0.489–0.785, *p* = 0.032). Based on the optimal cut‐off value of 307 ms (with the maximum Youden index), the sensitivity was 72.4% (22/31), specificity was 57.1% (16/28), positive predictive value (PPV) was 64.71%, and negative predictive value (NPV) was 64.0% (Figure 2A, Table 2). The results indicated that although the sensitivity of LVET as a single factor for diagnosing PE was acceptable, its specificity was low, suggesting that relying solely on LVET might lead to missed diagnosis in some cases.

**TABLE 2:** Diagnostic efficiency.

	Sensitivity	Specificity	PPV	NPV	AUC (*P*)
LVET	72.4%	57.1%	64.71%	64.0%	0.658 (0.032)
LVET+Hypertension	65.5%	77.8%	74.1%,	65.6%	0.776 (＜0.001)
LVET+Hypertension+HR	60.7%	82.1%	79.2%,	65.7%	0.769 (＜0.001)

Abbreviations: LVET, Left Ventricular Ejection Time; HR, Heart rates; PPV, positive predict value; NPV, negative predict value.

### 3.3 Multi‐Factor Combined Diagnostic Model

To improve the diagnostic efficacy, a multi‐factor logistic regression model was constructed.

(1) LVET combined with a history of hypertension: The AUC increased to 0.776 (95% CI: 0.661–0.891, *p* < 0.001), the sensitivity was 65.5% (20/31), the specificity was 77.8% (21/28), PPV was 74.1%, and NPV was 65.6% (Figure 2B, Table 2). Compared with LVET as a single factor, combining heart rate could improve diagnostic efficacy, and the increase in specificity might reduce false positives; however, the decrease in sensitivity might lead to missed diagnoses of false negatives.

(2) LVET combined with heart rate and history of hypertension: The AUC was 0.769 (95% CI: 0.652–0.886, *p* < 0.001), sensitivity was 60.7% (17/28), specificity was 82.1% (23/28), PPV was 79.2%, and NPV was 65.7% (Figure 2C, Table 2). Using the DeLong test to compare the differences in AUC between the two models, the results showed no statistical significance (*p* = 0.891), indicating that adding heart rate did not significantly improve the diagnostic efficacy. This might be related to the introduction of heart rate, which increased noise and affected the results; however, the increase in specificity might reduce false positives.

## 4 Discussion

PE, a multisystem dysfunction syndrome specific to pregnancy, has a complex pathophysiological mechanism involving interactions among multiple organs [14]. In recent years, with the development of noninvasive cardiovascular assessment techniques, the synchronized monitoring technology of heart sounds and electrocardiograms has gradually demonstrated its potential in the monitoring of cardiovascular function during pregnancy [15]. This study introduced the traditional cardiovascular parameter of LVET into the diagnostic system for PE, revealing its unique value in screening for PE. The research results showed that the LVET of pregnant women with PE was significantly prolonged (320 ± 26.79 ms vs. 301 ± 35.42 ms, *p* = 0.010), and the diagnostic efficacy significantly improved after combining with a history of hypertension (AUC = 0.776). This discovery not only provides a new idea for the early identification of PE, but also provides an important basis for an in‐depth understanding of the boundaries between the adaptive changes and pathological imbalance of cardiovascular function during pregnancy.

Early alterations in the vascular system have a significant predictive value in PE. This study found that the maximum amplitude of the second heart sound (Q2S2Max) was significantly elevated in the PE group (426.1 ± 29.46 mv vs. 403.96 ± 33.28 mv, *p* = 0.010). This parameter reflects the vibrational energy of the vascular wall in the aortic valve [13]. Its elevation is closely related to an increase in arterial stiffness and may result from increased collagen deposition and degradation of elastic fibers due to vascular endothelial dysfunction [16]. Clinical studies have shown that changes in arterial stiffness occur earlier than a significant increase in blood pressure, suggesting that Q2S2Max may serve as a sensitive indicator of early vascular dysfunction. This abnormal vascular‐cardiac coupling not only aggravates the afterload but may also participate in the process of myocardial ischemia by damaging coronary artery perfusion, forming a pathological triangle of “vascular injury—cardiac compensation—myocardial hypoxia” [17]. Therefore, changes in heart‐sound parameters provide a unique window for revealing the early compensatory mechanisms of the cardiovascular system in PE.

LVET, a key parameter for evaluating cardiac systolic function, profoundly reflects dynamic changes in the compensatory mechanism of the cardiovascular system for pathological load [18]. According to traditional hemodynamic theory, an increase in afterload is indicated by prolongation of isovolumic contraction time (IVCT) and shortening of ejection time, suggesting impaired left ventricular function [19]. For instance, in patients with ordinary hypertension, elevated peripheral vascular resistance leads to a significant shortening of LVET, which is closely related to a decline in myocardial contractile efficiency. However, this study found that patients with PE exhibited the opposite phenomenon of prolonged LVET (320 ± 26.79 ms vs. 301 ± 35.42 ms, *p* = 0.010), which contradicts the previous findings and suggests that its pathological mechanism is pregnancy‐specific, possibly involving a unique interaction between the placenta and the heart axis [20].

The unique physiological changes during pregnancy provide a structural basis for the prolongation of LVET. In normal pregnancy, a 40%–50% increase in blood volume leads to a significant increase in preload, which enhances myocardial contractility through the Frank‐Starling mechanism [21]. Systemic vasodilation begins in the 5th week of pregnancy and reaches its lowest point in the second trimester, reducing vascular resistance and accommodating the surge in blood volume. In this study, the mild increase in LVEF in the PE group (64.8 ± 3.5% vs. 62.8 ± 2.6%) may be partially attributed to this mechanism. Although afterload increases in PE, blood volume expansion maintains a higher ventricular filling pressure, allowing the left ventricle to maintain a longer effective working time during the ejection period. Computational fluid dynamics models show that geometric remodeling of the ventricle during pregnancy (such as chamber enlargement and ventricular wall thickening) can prolong LVET by approximately 8–12 ms under the same afterload, providing a biomechanical basis for explaining the changes in LVET in PE [22]. An imbalance in autonomic nerve regulation may be another key mechanism. Patients with PE often exhibit hyperactivity of the sympathetic nervous system, with plasma norepinephrine levels being 2‐3 times higher than those in normal pregnancies [23]. Sympathetic activation enhances myocardial contractility through β1 adrenergic receptors, allowing the left ventricle to eject more blood per unit time [24]. The prolongation of LVET in this study may reflect the compensatory prolongation of this positive inotropic effect; enhanced contractility accelerates the ejection rate, but the total ejection time is prolonged owing to the increased demand for each stroke volume. This compensation has a double‐edged sword effect: animal experiments have shown that continuous sympathetic activation can lead to desensitization of β receptors in the myocardium, ultimately resulting in depletion of contractile reserve [25]. This suggests that prolongation of LVET may be a transitional marker of compensation for decompensation, and its dynamic monitoring is of great significance for prognosis assessment.

Unlike heart failure with reduced ejection fraction (HFrEF), where reduced contractility and ventricular dilation shorten LVET [10], PE uniquely preserves LVET prolongation through compensatory mechanisms: sympathetic activation enhances β1‐adrenergic contractility, while retained blood volume (20%–30% above nonpregnant levels) sustains preload [24]. This adaptive response maintains stroke volume despite increased vascular resistance, distinguishing PE from HFrEF's decompensated state. LVET is emerging as a promising biomarker for PE due to its ability to reflect dynamic cardiovascular adaptations to placental and vascular pathology. In PE, elevated systemic vascular resistance (SVR) and endothelial dysfunction increase cardiac afterload, while compensatory mechanisms—such as sympathetic hyperactivity and retained blood volume—prolong LVET to maintain stroke volume. Unlike traditional biomarkers (e.g., sFlt‐1/PlGF ratio), LVET provides real‐time, noninvasive insights into cardiac workload and vascular‐cardiac coupling, enabling early detection of subclinical hemodynamic stress. By capturing both adaptive and maladaptive cardiac responses, LVET offers a unique window into PE pathophysiology, making it a practical and sensitive biomarker for risk stratification and intervention timing.

This study evaluated the diagnostic value of LVET using ROC curve analysis and explored the optimization path of the multi‐parameter combined model. The efficacy of LVET as an independent indicator has been demonstrated. The area under the curve of LVET for diagnosing PE was 0.658, with sensitivity and specificity of 72.4% and 57.1%, respectively. The high sensitivity suggests its suitability for the initial screening of high‐risk populations; however, the insufficient specificity indicates the need to combine other indicators. When the cutoff value was set at 307 ms, the negative predictive value (NPV) reached 64.0%, suggesting that LVET was normal, and the probability of excluding PE was relatively high. Through multi‐parameter combined modeling, diagnostic efficacy was significantly improved.

When LVET was combined with a history of hypertension, the AUC increased to 0.776 (95% CI: 0.661–0.891), and the specificity increased to 77.8%, with a positive predictive value (PPV) of 74.1%. A history of hypertension as an independent risk factor demonstrated its diagnostic value by identifying the tendency of vascular spasm, and the proportion of hypertension in the PE group was significantly higher than that in the control group (29.03% vs. 3.57%), while vascular spasm directly affected LVET by increasing peripheral resistance. This “functional parameter (LVET) + risk factor (hypertension history)” combination model captured the cardiac compensatory state and incorporated vascular pathological features, forming a multi‐dimensional risk assessment system. Further introduction of heart rate parameters increased the specificity to 82.1%, which might be related to the activation degree of the sympathetic nerve, as sympathetic tension increased, indirectly affecting ejection time by enhancing myocardial contractility, enabling the model to more accurately identify the high sympathetic activity subgroup.

Compared to traditional biomarkers, LVET has unique clinical application advantages. Urinary protein is a classic indicator for diagnosing PE, but its occurrence often lags behind cardiovascular function changes (in this study, 32% of PE patients had negative urinary protein at the first visit), and the false‐positive rate in normotensive pregnant women can reach 15% (such as pregnancy proteinuria). The sFlt‐1/PlGF ratio has excellent diagnostic efficacy (AUC > 0.9), but its detection relies on enzyme‐linked immunosorbent assay, with a single test cost exceeding 500 yuan and a time consumption of up to 4–6 h, making it difficult to meet the dynamic monitoring needs of grassroots medical care [26]. In contrast, LVET has a lower detection cost and can be obtained in real time, making it a suitable screening tool. Additionally, the consistency between LVET and ultrasound EF supports the formation of complementarity between the two and provides precise structural and functional assessment, while heart sound and electrocardiogram technology achieve dynamic tracking, especially in resource‐limited areas, where the combination of the two can build a stratified diagnosis and treatment system–LVET abnormal patients are prioritized for ultrasound diagnosis.

The integration of ECG‐PCG technology into prenatal care holds significant promise for resource‐limited settings such as rural Xinjiang. Our wearable device (98 g, 6 × 4 cm) enables continuous monitoring ( > 24 h) with minimal training—nurses achieved 92% interpretation accuracy after a 2‐h session. Field testing in Xinjiang clinics demonstrated a 98% operational uptime over 6 months, with an annual recalibration cost of 2000 yuan per device. At a per‐test cost of 150 yuan (vs. 500yuan for sFlt‐1/PlGF), this approach reduced echocardiography referrals by 40% in pilot studies, highlighting its potential to alleviate healthcare disparities.

This study has several limitations. First, the sample size was relatively small (*n* = 59) and was based on data from a single center, which may affect the generalizability of the results. Second, data on the dynamic changes in LVET (such as at different gestational weeks and circadian rhythms) were not included, making it difficult to reveal its longitudinal predictive value. Third, our study cohort was drawn from Xinjiang, a region with significant ethnic diversity (e.g., Uyghur, Han, Kazakh). While this reflects real‐world clinical diversity, genetic and epigenetic variations across ethnic groups may influence LVET's predictive performance. For instance, Uyghur women have a 1.3‐fold higher incidence of PE compared to Han women in Xinjiang, potentially due to differences in placental angiogenesis pathways. Future studies should stratify analyses by ethnicity to refine generalizability. Finally, 10.2% of the heart‐sound signals needed to be re‐collected due to fetal movement and changes in the pregnant woman's body position. Future efforts should be made to optimize signal processing algorithms (such as AI‐assisted artifact recognition).

## Author Contributions

Qiong Huang, Ningtian Zhou, Xinxin Zhang, and Chunping Tang: Designed, supervised the study and prepared the manuscript. Yiyuan Xiong, Yingxia Zhu, and Ningtian Zhou: Data collection and analysis, writing original draft. Chunping Tang, Xinxin Zhang, and Maio Wang: Data collection and analysis. Miao Wang, Ningtian Zhou, and Qiong Huang: Writing – Review and Editing. Xinxin Zhang, Ningtian Zhou, Chunping Tang, and Qiong Huang: Project administration and data analysis.

## Conflicts of Interest

The authors declare no conflicts of interest.

## Data Availability

The data that support the findings of this study are openly available in DOI: 10.17632/mrwv6g3gtt.1.
